# Can the duration of tuberculosis treatment be shortened with higher dosages of rifampicin?

**DOI:** 10.3389/fmicb.2015.01117

**Published:** 2015-10-14

**Authors:** Noton K. Dutta, Petros C. Karakousis

**Affiliations:** ^1^Department of Medicine, Center for Tuberculosis Research, Johns Hopkins University School of MedicineBaltimore, MD, USA; ^2^Department of International Health, Johns Hopkins Bloomberg School of Public HealthBaltimore, MD, USA

**Keywords:** *Mycobacterium tuberculosis*, rifampicin, treatment improvement, persistence, reactivation, animal models, clinical trial

## Introduction

Strategies involving new applications of existing drugs are urgently needed to reduce the time required to cure patients with drug-susceptible and drug-resistant tuberculosis (TB). Use of high-dosage rifampicin is one such approach. Recent data from preclinical animal models (Hu et al., 2015) and clinical studies (Boeree et al., 2015) support a potential role for high-dosage rifampicin in TB chemotherapy, although more studies are required to guide optimal clinical management. Specifically, further basic preclinical research is needed to: (i) Develop pharmacokinetic/pharmacodynamic models to improve our understanding of drug bioavailability and activity in tissues; (ii) Determine the antimicrobial efficacy and the ability of high-dosage rifampicin to reduce the emergence of antibiotic resistance; and (iii) Explore high-dosage rifampicin for evaluation of new combination regimens to achieve the ultimate goal of shortening TB treatment and achieving stable cure without relapse.

## An update on the use of high-dosage rifampicin for the treatment of tuberculosis

### Rifampicin and its role in dots (directly observed treatment, short-course)

Combination chemotherapy has been the standard of care for TB since the 1950s, when it was demonstrated that combining streptomycin with para-aminosalicylic acid and, later, with isoniazid prevented the emergence of drug resistance and enabled reliable cures following 18–24 months of treatment (1972). In the 1980 s, the sequential incorporation of rifampicin and pyrazinamide accelerated the eradication of bacterial “persisters” and shortened the duration of treatment needed to prevent relapse. Combining the synergistic antimicrobial properties of rifampicin and pyrazinamide with the potent bactericidal activity of isoniazid formed the basis of the current 6-month “short-course” regimen, which showed the least frequency of relapse. Yet, despite the high efficacy of this treatment and efforts to implement it throughout the world, TB remains a global health emergency, in part because even a 6-month regimen poses formidable challenges for the resource-limited healthcare infrastructures of many TB-endemic countries.

### Mechanism of action and resistance

Rifampicin is a semisynthetic derivative of rifamycin B which is produced by *Amycolatopsis* (formerly *Streptomyces*) *rifamycinica*. It is one of the key drugs for the short course TB regimen and possesses bactericidal as well as sterilizing activity against tubercle bacilli in both cellular and extracellular locations. The rifamycins are highly protein-bound in plasma, but easily diffuse across the *Mycobacterium tuberculosis* cell envelope due to their lipophilic nature (Wade and Zhang, 2004). The bactericidal activity of the rifamycins is attributed to their ability to inhibit transcription by binding with high affinity to bacterial DNA-dependent RNA polymerase (Hartmann et al., 1967; Jin and Gross, 1991; Campbell et al., 2001).

The development of rifampicin resistance is strongly associated with lower serum drug concentrations (Pasipanodya et al., 2013), while higher maximum concentration (C_max_) and area under the serum concentration-time curve (AUC_0−24_) inhibit the development of rifampicin resistance (Gumbo et al., 2007). Mutations in the *rpoB* gene account for over 95% of clinical cases of rifampicin resistance and are commonly associated with the presence of multidrug-resistant tuberculosis (Shah et al., 2007). Unlike mutations in codons 531 and 526, which confer high-level resistance to rifampicin (MIC > 32 μg/ml) and cross-resistance to all rifamycins (Wade and Zhang, 2004), mutations in codons 511, 516, and 522 are associated with low- or high-level resistance to rifampicin (MIC 2–32 μg/ml) (Bodmer et al., 1995; Moghazeh et al., 1996; Williams et al., 1998).

Consistent with the clinical data, selection of spontaneous rifampicin resistance *in vitro* in the *M. tuberculosis* laboratory reference strain H37Rv indicate that the Ser_531_-to-Leu mutation and multiple mutations in codon 526 occur at a significantly higher frequency than other point mutations (Billington et al., 1999). In fact, mutants with low-level rifampicin resistance appear to be better adapted to *in vivo* growth than mutants with high-level rifampicin resistance. Mutant strains can be enriched in the presence of drug pressure (Mariam et al., 2004). Whether higher dosages of rifampicin facilitate the emergence of mutants with higher MIC of the drug requires further study. Louw et al. showed that the level of rifampicin resistance is determined by the activation of efflux and transporter genes (Louw et al., 2011). Recent data suggest that in addition to classical mutations in *rpoB*, the efflux pumps Rv2333, DrrB, DrrC, Rv0842, BacA, and EfpA may have a role in rifampicin resistance (Li et al., 2015).

### Pharmacokinetics and pharmacodynamics

Rifampicin is used at a dose of 600 mg per day throughout the 6-month TB treatment course. This dosing scheme was determined in the 1960 s based on cost and efficacy, although the highest tolerable dose was not defined (Steingart et al., 2011; van Ingen et al., 2011). Rifampicin's microbial killing was linked to the area under the AUC_0−24_/minimum inhibitory concentration (MIC) ratio (Jayaram et al., 2003). However, many patients achieve rifampicin AUC_0−24_/MIC and C_max_/MIC ratios associated with suboptimal microbial killing and resistance suppression (Peloquin et al., 1997), indicating that higher dosages of rifampicin could improve treatment outcomes, so long as patients can tolerate them. Following delivery of the standard 600-mg dosage, rifampicin concentrations attained at the site of infection were determined to be too low (Ziglam et al., 2002; Goutelle et al., 2009). Interestingly, the use of a 1200-mg rifampicin dosage significantly increased the probability of attaining AUC_0−24_/MIC or C_max_/MIC ratios compatible with bacterial killing (Goutelle et al., 2009). Recent studies indicate that increases in rifampicin dosage result in concentrations that are more than dose-proportional; specifically, a 2-fold increase in dosage from 10 to 20 mg/kg daily results in a 4-fold increase in the AUC_0−24_ and enhanced early bactericidal activity for each increase in dosage (Boeree et al., 2015). In addition, higher dosages of rifampicin may result in nonlinear increases in drug concentrations inside the bacteria, a phenomenon possibly related to saturation of bacterial efflux pumps (Gumbo et al., 2007). Piddock et al. demonstrated the accumulation of rifampicin by mycobacteria in the presence of efflux inhibitor reserpine (Piddock et al., 2000). There is a need to assess whether high-dosage rifampicin affects the pharmacokinetics of other anti-TB drugs and antiretroviral drugs, particularly its inductive effect on the cytochrome P450 enzyme system. While physiologically based pharmacokinetic modeling is often motivated by animal-to-human scaling (Savic et al., 2014), the differences in variability, absorption, distribution, metabolism, and excretion of rifampicin between mice and humans should be considered (Lyons et al., 2013). For example, penetration of rifampicin into lung cavities cannot be modeled in standard mouse models of TB, which lack cavities (Lenaerts et al., 2015). The rate of intestinal absorption of rifampicin is reduced in mice when given at higher dosages, although this does not affect total AUC. The impact of higher protein binding in mice (96%) relative to human (89%) on differences in pharmacokinetic and pharmacodynamic properties of the drugs is unknown. In addition, rifampicin can reduce the plasma concentrations of drugs that are not metabolized (e.g., digoxin) by inducing drug transporters such as P-glycoprotein. Finally, mice do not generate 25-desacetyl rifampicin, which is the main metabolite of rifampicin in humans (Wilkins et al., 2008; Dutta et al., 2012, 2013).

### Recent advances

Recently, existing drugs are being repurposed or optimized for TB with the goal of shortening the duration of treatment for drug-sensitive and drug-resistant TB. Use of high-dosage rifampicin is one such approach. Results from studies with mice (Jayaram et al., 2003) and early bactericidal activity studies (Diacon et al., 2007) indicate that a single dosage of 600 mg of rifampicin in TB treatment is at the lower end of the concentration-response curve. Using the murine TB model, Rosenthal et al. showed that increasing the dosage of rifampicin significantly increased the sterilizing activity of the regimen (Rosenthal et al., 2012). Steenwinkel et al. reported that an eightfold increase in the currently used 10 mg/kg rifampin dosage was well tolerated and allowed reduction of therapy duration from 6 to 2 months (de Steenwinkel et al., 2013). The study by Hu et al. (2015) investigated the role of high-dosage rifampicin against *Mycobacterium tuberculosis* persisters in an *in vitro* model of progressive hypoxia and in the Cornell mouse model of persistence. The authors found a dose-proportional increase in rifampicin C_max_ and AUC_0−24_, resulting in eradication of resuscitation promoting factor-dependent persisters. Hu et al. study also showed that lung culture-conversion and relapse-free cure were obtained much earlier in mice treated with high-dosage rifampicin (50 mg/kg) (Hu et al., 2015), thereby allowing for an abbreviated treatment course without incurring disease relapse. It has been poignantly recognized in the TB field recently that observations made in mice are not necessarily predictive of outcomes in human clinical trials of TB chemotherapy, nor is early “sterilization” a predictor of cure (Gillespie et al., 2014).

Recently, data from patients with osteoarticular tuberculosis suggest that increasing the rifampicin concentration at the site of infection may optimize this drug's antitubercular effect, even against some rifampicin-resistant isolates, if systemic toxicity can be minimized (Zhang et al., 2014). Historical trials suggest that higher than standard rifampicin dosing results in improved culture conversion rates (please see systematic review (Steingart et al., 2011). Currently, several clinical trials are examining the efficacy and safety of higher dosages of rifampicin than the currently used dosage of 10 mg/kg against drug-susceptible TB. Phase II and III clinical trials evaluating higher dosages of rifampicin and other rifamycins are needed to confirm efficacy and assure tolerability. PanACEA (the Pan-African Consortium for the Evaluation of Antituberculosis Antibiotics) which is funded by the European and Developing Countries Clinical Trials Partnership, has found that administering up to 35 mg/kg of rifampicin is safe and well tolerated, resulting in a non-linear increase in exposure to rifampicin without an apparent ceiling effect, and increased early bactericidal activity at 14 days (HIGHRIF1-phase IIA, multiple dose rising study grouping 20, 25, 30 up to 35 mg RIF/kg, NCT01392911); (Boeree et al., 2015). However, it is important to note that as we are dealing with months of exposure to rifampin, a drug administration of 2 weeks only qualifies as an acute toxicity study and cannot provide sufficient estimation of toxicities from subacute or chronic exposure. A second trial (HIGHRIF2- phase IIb, NCT00760149) is examining the efficacy of rifampicin given at 10, 15, and 20 mg/kg daily. Although the microbiological data are not yet available, this study found no serious adverse events for 2 months of rifampicin at 15 and 20 mg/kg. HIGHRIF3 (phase II) is a dosage-ranging study designed to identify the optimal rifampicin dosage for evaluation of efficacy. The group recently started the above-described multi-arm multi-stage study, which tests one group with 35 mg/kg of rifampicin (isoniazid/rifampin_35_/pyrazinamide/ethambutol), a second group with 20 mg/kg of rifampicin combined with moxifloxacin (isoniazid/rifampicin_20_/pyrazinamide/moxifloxacin), and a third with 20 mg/kg of rifampicin combined with the novel ethylenediamine, SQ109. Preliminary analysis of the data suggests that the first two groups may shorten the duration of TB treatment. The International Consortium for Trials of Chemotherapeutic Agents in Tuberculosis (INTERTB) will soon publish the results of the RIFATOX study, which indicated that rifampicin at 900 and 1200 mg daily for the first 4 months of the standard 6-month regimen was safe, with no increase in serious adverse events (ISRCTN55670677). However, these higher dosages of rifampicin did not significantly improve culture conversion rates at 2 months. Based on these results, a phase III study (RIFASHORT) has been initiated to assess the treatment-shortening potential of high-dosage (1200/1800 mg) rifampicin (2 months of isoniazid/rifampicin/pyrazinamide/ethumbutol + 4 months of isoniazid/rifampicin). The NIAID HIRIF study, started in September 2013, is a randomized trial of high-dosage rifampicin in patients with new, smear-positive TB (NCT01408914). French National Institute for Health and Medical Research-French National Agency for Research on AIDS and Viral Hepatitis (Inserm-ANRS) started RIFAVIRENZ (NCT01986543), a drug-drug interaction study between high dosage rifampicin and efavirenz in the context of pulmonary tuberculosis and HIV co-infection. Recent report suggests that rifampicin and rifapentine significantly reduce concentrations of bedaquiline, a new anti-TB drug (Svensson et al., 2015).

## Conclusions

Use of high-dosage rifampicin against *Mycobacterium tuberculosis* is promising as it may not only result in enhanced killing of mycobacteria and shorter therapy duration, but may also result in prevention of drug resistance (Gumbo et al., 2007; Goutelle et al., 2009; Rosenthal et al., 2012; de Steenwinkel et al., 2013; Boeree et al., 2015; Hu et al., 2015), which are highly desirable properties in an anti-TB regimen. While these preclinical studies are subject to questions regarding their predictive accuracy for assessing the efficacy of anti-TB regimens, the results of ongoing phase IIB studies promise to provide further guidance on the optimal dosage of rifampin (Boeree et al., 2015). However, whether high-dosage rifampicin results in reduced relapse rates remains to be explored in clinical studies. Additional preclinical research using well-validated animal models of tuberculosis is warranted to guide the future study of high-dosage rifampicin in clinical trials. For joint HIV–TB treatment, it is important to determine if high-dosage rifampicin can shorten the time required to cure TB without increasing adverse events and drug interactions with other antitubercular drugs and antiretroviral agents.

## Funding

This study was funded in part by National Institutes of Health grant R01AI106613 (to PK) and by the Johns Hopkins University Center for AIDS Research Tangible Research Initiative award (to ND).

### Conflict of interest statement

The authors declare that the research was conducted in the absence of any commercial or financial relationships that could be construed as a potential conflict of interest.
